# ASICs may affect GABAergic synapses

**DOI:** 10.18632/oncotarget.18247

**Published:** 2017-05-28

**Authors:** Maksim Storozhuk, Oleg Krishtal

**Affiliations:** A.A. Bogomoletz Institute of Physiology, Kiev, Ukraine

**Keywords:** ASICs, synaptic transmission, amiloride, diminazene, GABA_A_ receptors

Currents activated in the neuronal membrane by extracellular acidification were first reported in 1980 [1]. The existence of specific proton-activated receptor was postulated. In 1997 this receptor was cloned and named “acid-sensing ion channel”, ASIC [2]. There are at least six different ASIC subunits (ASIC1a, ASIC1b, ASIC2a, ASIC2b, ASIC3, and ASIC4) coded by four different genes. All ASICs belong to the degenerin/epithelial Na+ channel (DEG/ENaC) superfamily, which comprises Na^+^-selective cation channels. Functional ASICs assembled in plasma membrane are homo- or heteromeric trimers representing channels with distinct properties. ASICs are widely expressed in both peripheral and central nervous system [3] and play a role in a variety of important physiological functions and pathological states, including oncological [4]. Thus, it is not surprising that ASICs are considered a promising pharmacological target.

Although under some pathological conditions ASICs can be persistently active (even in the absence of protons) [6], it is important to mention that typically these channels are activated by a rapid increase in the concentration of extracellular protons and rapidly desensitize/inactivate after proton-induced activation. Slow acidification results in a steady-state desensitization. Thus, there are actually very few places in the brain where ASICs can be activated under physiological conditions. Synaptic cleft is one of them. Rapid acidification occurring during synaptic vesicles release [5] can activate ASICs both on pre- and postsynaptic neurons. In the latter case, a fraction of postsynaptic current would be mediated by ASICs with protons acting as a co-transmitter. This has been recently reported for gluatamatergic synapses in amygdala [6]. Additionally, activation of ASICs could modulate synaptic strength by affecting transmitter release and/or sensitivity of postsynaptic receptors.

We examined these possibilities in relation to hippocampal GABAergic synapses [7]. Given that the endogenous acidification of the synaptic cleft is sufficient to activate ASICs and they play a functional role in a particular type of synapses, it would be expected that blockers of ASICs affect synaptic currents.

In this regard, we studied effects of three structurally different blockers of ASICs (compound 5b, amiloride and diminazene) on GABAergic postsynaptic currents (PSCs). We found that GABAergic (PSCs), recorded as inward currents (that is, at membrane voltages more negative than the reversal potential), were suppressed by all the employed blockers of ASICs. In the same cells the suppression of PSCs, recorded as outward currents (above the reversal potential) was statistically insignificant. These results imply that the effects of blockers in our experiments are at least partially postsynaptic. On the other hand, in the case of mediation of a fraction of PSCs by ASICs, an increase of outward currents would be expected under our experimental conditions. Moreover, in the case of mediation of a fraction of PSCs by ASICs, an enhancement of the effects of the blockers of ASICs on PSCs would be expected when GABA_A_-receptors are blocked. However, a fraction of postsynaptic current resistant to the blocker of GABA_A_-receptors bicuculline was not substantially affected by ASICs blocker (compound 5b).

Altogether, our data imply that ASICs are involved in modulation of GABAergic postsynaptic currents. Occam’s razor allows suggesting that this modulation occurs via interaction of activated ASICs with GABA_A_ receptors.

It should be mentioned that the search for modulatory role of ASICs in regulation of GABAergic transmission (in spite the above mentioned results of experiments with bicuculline) was advised by Dr. Irina Vladimirova to M.S. (personal communication). This helped us a lot in keeping trying and finally revealing a modulatory role of ASICs.

In conclusion, our results indicate that ASICs play a functional role at hippocampal GABAergic synapses, which is so far unusual. Still, functional crosstalk between ASICs and GABA_A_ receptors has been previously reported in isolated neurons [8].

The reason why we did not reveal ASICs-mediated current in this synaptic system may be due to the small absolute and relative amplitude of ASICs-mediated current (see [7] for details).
